# Supplementary material for: Effect of intrapartum azithromycin on early childhood gut mycobiota development: post hoc analysis of a double-blind randomized trial
Source: Nat Commun. 2025 Aug 9;16:7356. doi: 10.1038/s41467-025-62142-w (PMC12335549; doi:10.1038/s41467-025-62142-w)
Supplement: Supplementary file 1 — Supplementary Information [file 41467_2025_62142_MOESM1_ESM.pdf]

Effect of intrapartum azithromycin on early childhood gut mycobiota development: post hoc analysis of a double-blind randomized trial

Supplementary Table 1. Sample counts before and after filtering

	Initial number of samples, *N*		Samples removed by filter, n (%*N*)		Final number of samples, n (%*N*)	
Time-point	Azithromycin	Placebo	Azithromycin	Placebo	Azithromycin	Placebo
day 0	43	50	8 (18.6%)	4 (8.0%)	35 (81.4%)	46 (92.0%)
day 6	40	48	2 (5.0%)	5 (10.4%)	38 (95.0%)	43 (89.6%)
day 28	46	53	5 (10.9%)	7 (13.2%)	41 (89.1%)	46 (86.8%)
month 4	45	50	1 (2.2%)	6 (12.0%)	44 (97.8%)	44 (88.0%)
year 3	40	52	1 (2.5%)	2 (3.8%)	39 (97.5%)	50 (96.2%)
Total	214	253	17 (7.9%)	24 (9.5%)	197 (92.1%)	229 (90.5%)

Note: samples removed by filter are samples that dropped out due to filtering of human, unclassified and contaminant reads.

Supplementary Table 2. list of taxa that were identified as contaminants using decontam. Both the frequency and prevalence methods were applied in combined mode. P-values were adjusted for multiple testing using Benjamini-Hochberg method.

Taxid	freq	prev	p.freq	p.prev	p	contaminant
Saccharomyces cerevisiae	0.42253763903732400	468	0.9669420592332160	2.23056441464633E-04	0.0020364114443620100	TRUE
Saccharomyces	0.05042749077679010	351	0.9999983642759590	1.03785107700519E-09	2.25069211693592E-08	TRUE
Botrytis cinerea	0.011082249758460300	146	0.8764545761725360	9.5272054465775E-16	2.98260137251679E-14	TRUE
Fusarium oxysporum	0.004314569111632140	77	0.2680972768966100	1.69570830602657E-18	1.96551956659766E-17	TRUE
Saccharomycetales	0.005068043744929240	79	0.925672392690078	1.70721956777188E-14	5.18008115467327E-13	TRUE
Ascochyta rabiei	0.004631845784211130	71	0.6914122975042120	0.03135990011706060	0.1047540356968130	TRUE
Aspergillus chevalieri	0.004136326140866770	38	0.5945998945185460	0.02531911593664990	0.07822538364274240	TRUE
Sordariomycetes	0.007557768574684600	126	0.7913842088794870	2.5621425810791E-30	1.40658709977184E-28	TRUE
Aspergillus	0.0024214659861353400	10	0.36652674970803300	0.08166795268986280	0.13496343935796500	TRUE
Tetrapisispora blattae	0.018878357565908900	179	0.6264898653641240	2.55257443769222E-33	1.22361206251962E-31	TRUE
Fulvia fulva	0.004829026566709900	76	0.6454723426879810	1.23285241276152E-11	2.11332238402859E-10	TRUE
Debaryomycetaceae	0.0013114947382284100	28	0.7172684693381460	5.96696537992962E-04	0.003747668734051560	TRUE
Aspergillaceae	0.0018195072295135300	12	0.4172345821674800	0.049108711830383800	0.10015082526089300	TRUE
Fusarium sambucinum species complex	0.001052040921522930	27	0.8589479994925700	5.83233576189704E-07	7.76836235474005E-06	TRUE
Talaromyces	1.44430851550352E-04	2	0.04459662580861100	0.6842653802632360	0.13700145409601700	TRUE
Colletotrichum lupini	9.28538779611212E-04	31	0.8574207975634120	1.7677669410238E-07	2.53158684856939E-06	TRUE
Fusarium graminearum	0.0054134615343052100	118	0.4118268880317610	5.69040435124816E-30	1.62228288011485E-28	TRUE
Fusarium fujikuroi	2.75307445075766E-04	8	0.5236924660929780	0.03546882363930810	0.09261283745424760	TRUE

Supplementary Table 3. Change in Shannon diversity by age within each trial arm assessed using a linear mixed effects model, adjusted by season of sample collection, mother's ethnicity, parity, and sex. Random variations were averaged on individuals to control for repeated measurements. Exact p-value for day 0 vs day 6 in azithromycin arm = 0.0003

Interval	Azithromycin p-value	Placebo p-value
day 0 to day 6	<0.001	0.997
day 0 to day 28	0.360	0.212
day 0 to month 4	0.008	0.283
day 0 to year 3	0.027	0.281

Supplementary Table 4. Community composition (beta-diversity) by age, season of sample collection, ethnicity, parity, and sex within each trial arm estimated using PERMANOVA with permutations restricted within individuals to control for repeated measurements.

	Azithromycin		Placebo	
Variable	R^2	p-value	R^2	p-value
Age	0.071	0.002	0.081	<0.001
Season of sample collection	0.012	0.164	0.012	0.166
Ethnicity	0.039	0.111	0.027	0.057
Parity	0.009	0.143	0.009	0.525
Sex	0.010	0.815	0.007	0.055

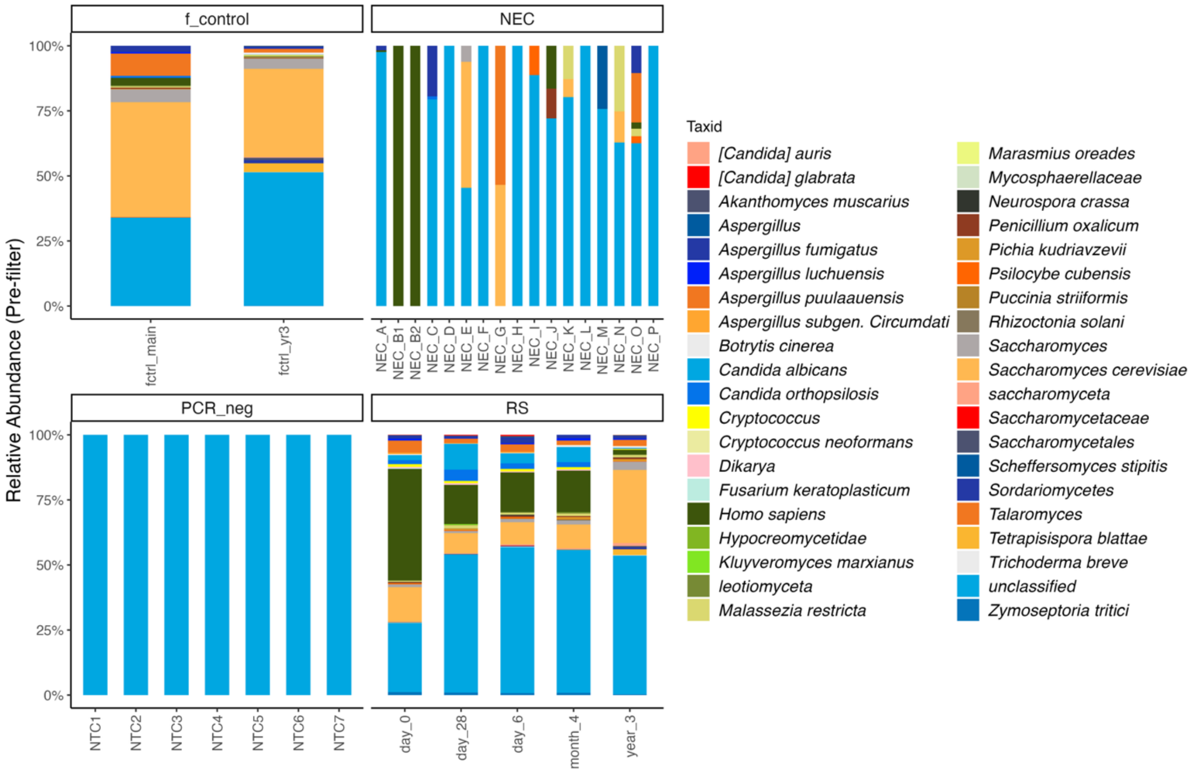

Supplementary Figure 1. Initial profiles of samples and controls before any filtering. Most reads in PCR and extraction blanks (NEC_*) are unclassified. Most reads in the field controls are *Saccharomyces cerevisiae* and unclassified. As expected, there are more human reads in day_0 samples than those from the subsequent time-points (day 0 = 39.6%, day 6 = 13.2%, day 28 = 14.8%, month 4 = 15.1%, year 3 = 1.6%). f_control = field control, NEC = negative extraction control (extraction blank), PCR_neg = PCR negative (PCR blank), RS = rectal swab

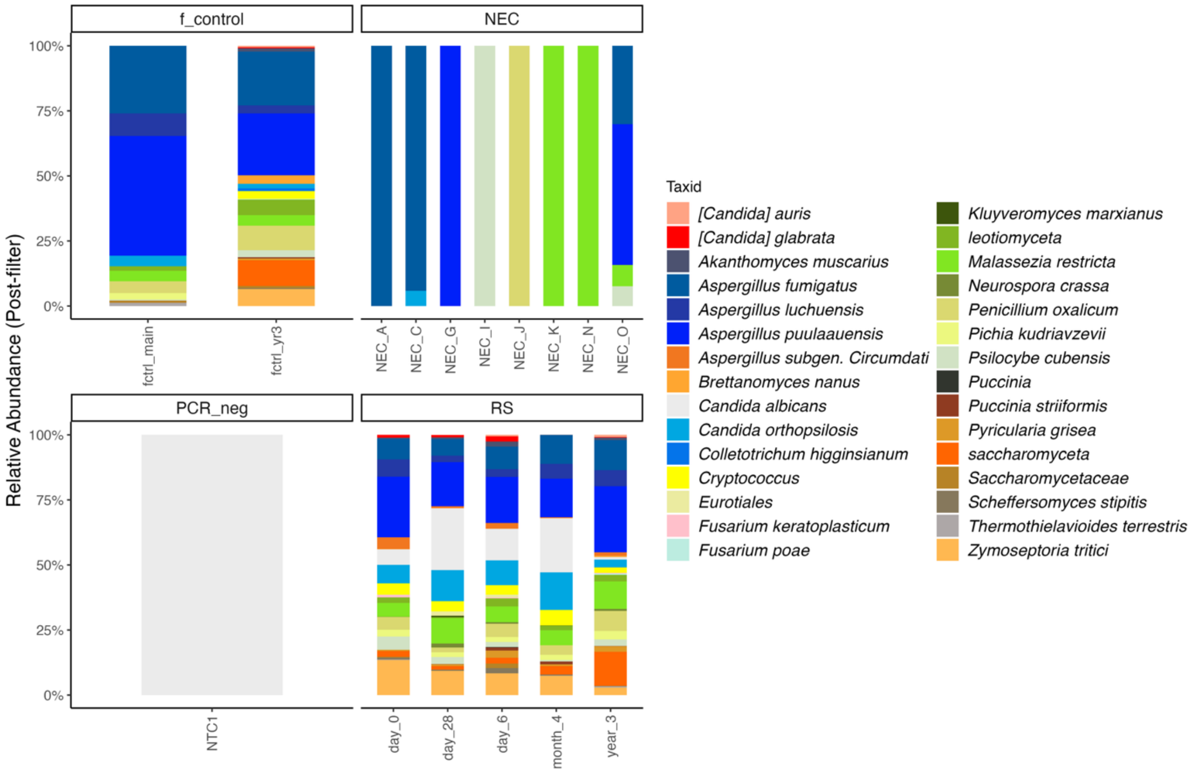

Supplementary Figure 2. Profiles of samples and controls after filtering human and unclassified reads and contaminants identified with decontam. There are two main taxa remaining in the field controls which may be from the STGG or environment. They are *Aspergillus fumigatus* and *Aspergillus puulaauensis*, which were filtered from the dataset. f_control = field control, NEC = negative extraction control (extraction blank), PCR_neg = PCR negative (PCR blank), RS = rectal swab

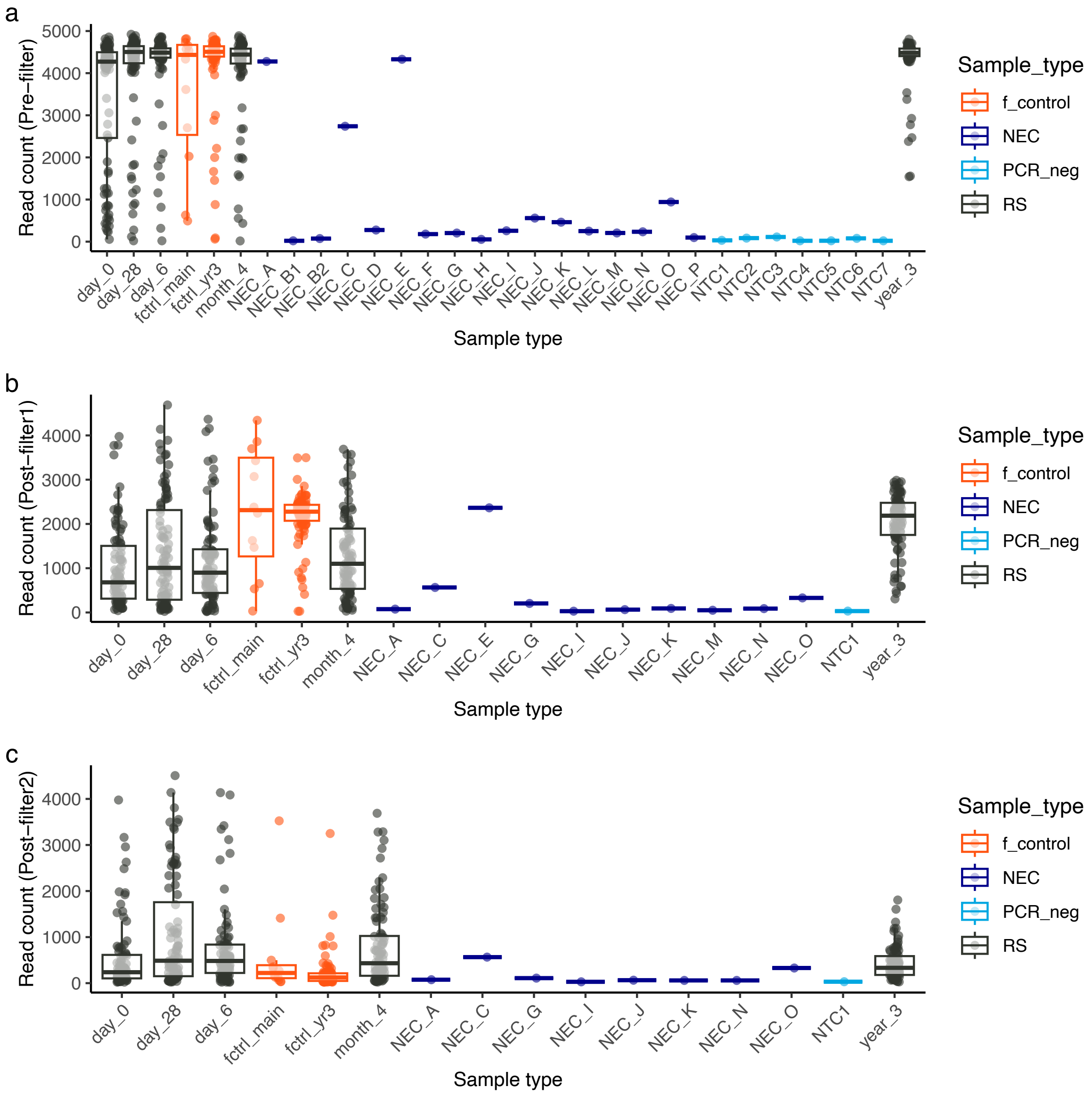

Supplementary Figure 3. Read counts of samples and controls before and after filtering. The box and whiskers denote the distribution of read counts. The box shows the median and lower and upper quartiles (middle 50% of read counts), while the whiskers show the upper and lower 25% read counts including the maximum and minimum values. (a) read counts before any filter was applied. (b) read counts after removing human and unclassified reads. (c) read counts after removing contaminants identified with decontam. f_control = field control, NEC = negative extraction control (extraction blank), PCR_neg = PCR negative (PCR blank), RS = rectal swab

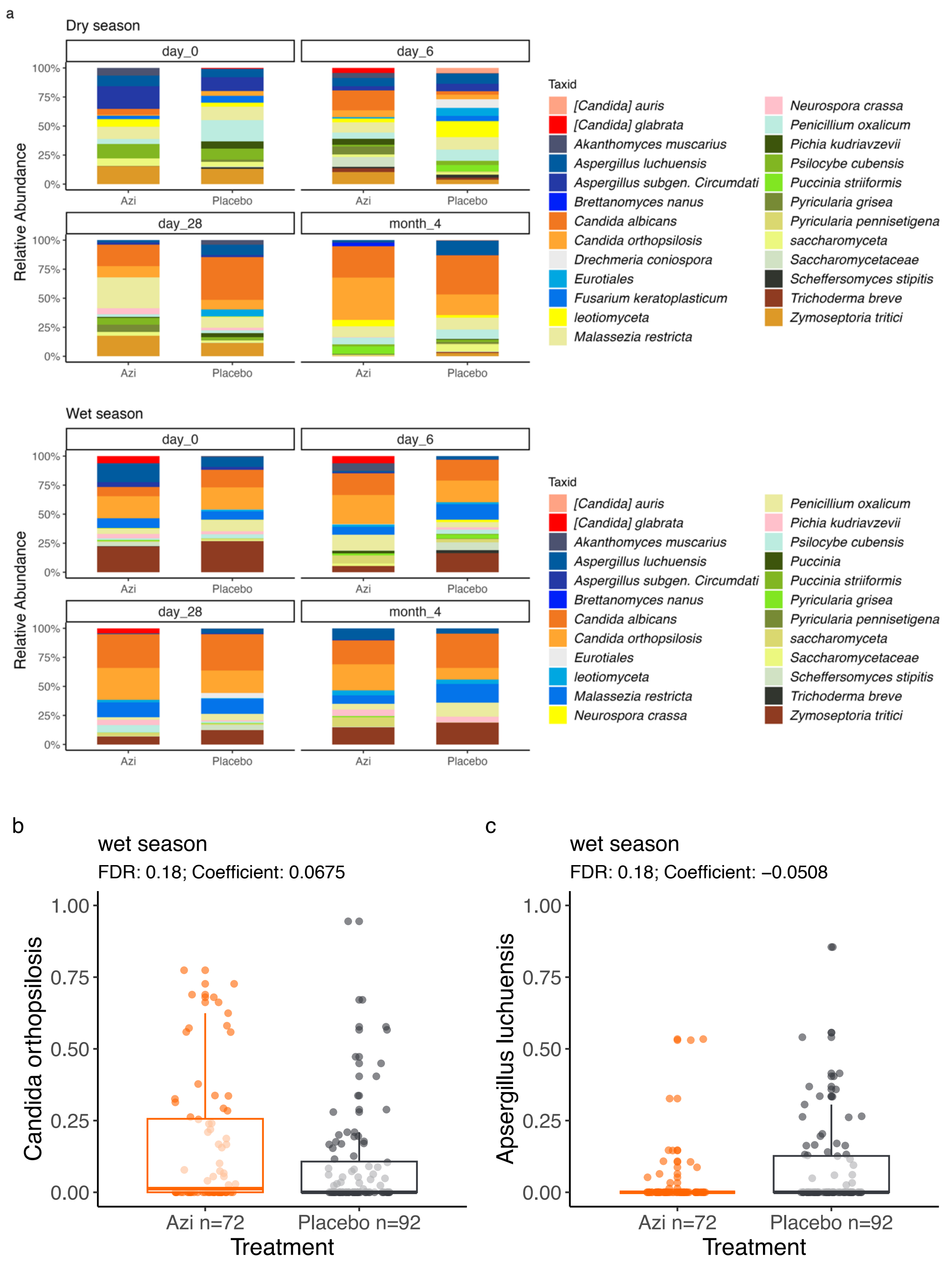

Supplementary Figure 4. Community profiles and differential taxon abundance between trial arms by season. a) Community profiles compared between trial arms at each time-point by season. b) Abundance of *C. orthopsilosis* between trial arms in the wet season. c) Abundance of *A. luchuensis* between trial arms in the wet season. Differential abundance was estimated

using MaASLin2. The box and whiskers show the distribution of taxon relative abundance in individual samples. The box shows the median and lower and upper quartiles (middle 50% of the relative abundances), while the whiskers show the upper and lower 25% relative abundances including the maximum and minimum values. Azi = Azithromycin

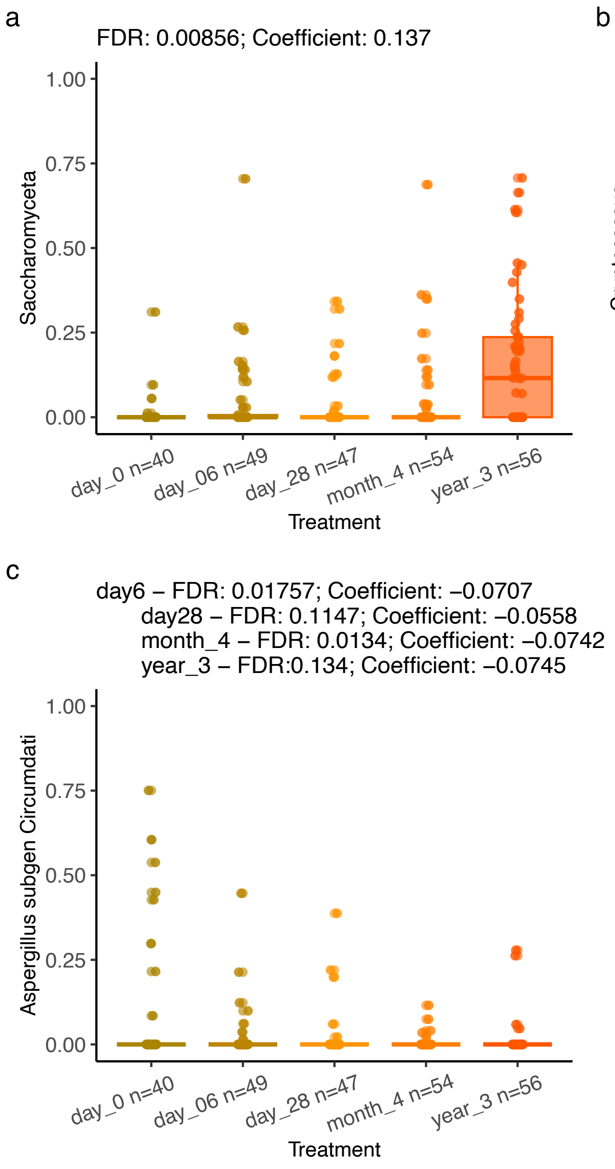

Supplementary Figure 5. Differential taxon abundance by age estimated using MaASLin2. The reference group for age comparisons is day 0 (day of birth). a) Abundance of *Saccharomyceta* is higher at year_3. b) Abundance of *Cryptococcus* is lower at year_3. c) Abundance of *Aspergillus subgenus Circumdati* is lower at all time-points after day_0. The box shows the median and lower and upper quartiles (middle 50% of the relative abundances),

while the whiskers show the upper and lower 25% relative abundances including the maximum and minimum values.

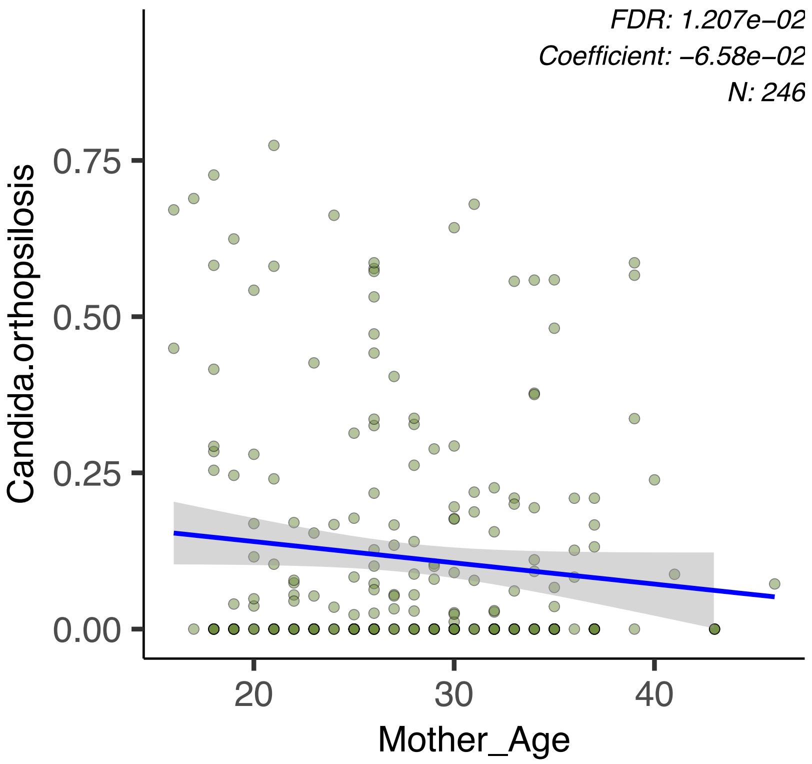

Supplementary Figure 6. Correlation of maternal age with abundance of *Candida orthopsilosis*. Abundance decreases with increasing age.

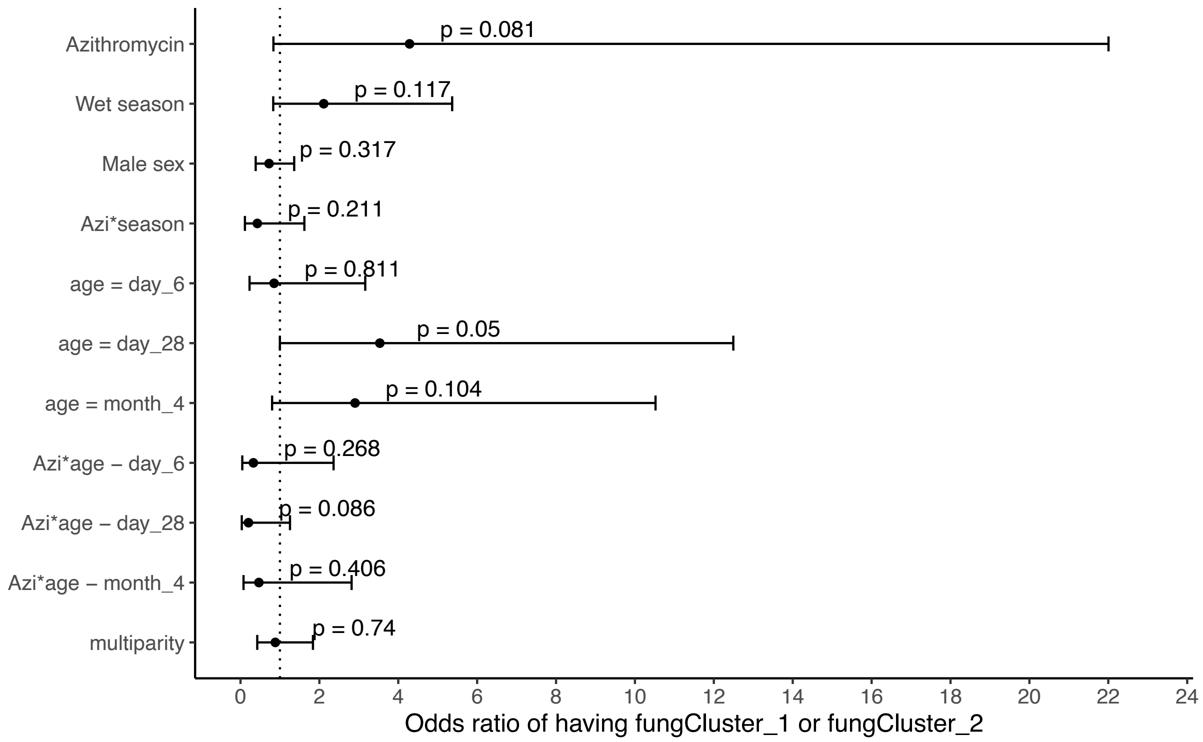

Supplementary Figure 7. Association of fungal community types with mycobiota covariates by logistic regression using a generalised linear model (N = 191 samples). References for comparisons: Treatment: placebo, Sampling season: dry season, Sex: female, Age: day_0, Parity: primipara. Error bars indicate mean and 95% confidence interval of odds ratio of having fungCluster_1 or fungCluster_2. Azi = Azithromycin
