# Supplementary material for: Effect of intrapartum azithromycin on early childhood gut mycobiota development: post hoc analysis of a double-blind randomized trial
Source: Nat Commun. 2025 Aug 9;16:7356. doi: 10.1038/s41467-025-62142-w (PMC12335549; doi:10.1038/s41467-025-62142-w)
Supplement: Supplementary file 2 — Description of Additional Supplementary Files [file 41467_2025_62142_MOESM2_ESM.pdf]

File Name: Supplementary Data 1

Description: Bacterial community-types (clusters) assigned to individual samples based on community similarity determined by Dirichlet's multinomial mixtures model implemented in mothur. This data was generated in our previous analysis on the effect of intrapartum azithromycin on gut microbiota development using the same samples in the current analysis.
