# Supplementary material for: Effect of intrapartum azithromycin on early childhood gut mycobiota development: post hoc analysis of a double-blind randomized trial
Source: Nat Commun. 2025 Aug 9;16:7356. doi: 10.1038/s41467-025-62142-w (PMC12335549; doi:10.1038/s41467-025-62142-w)
Supplement: Supplementary file 4 — Reporting Summary [file 41467_2025_62142_MOESM4_ESM.pdf]

nature portfolio

Corresponding author(s): Anna Roca
Last updated by author(s): Jul 7, 2025

Reporting Summary

Nature Portfolio wishes to improve the reproducibility of the work that we publish. This form provides structure for consistency and transparency in reporting. For further information on Nature Portfolio policies, see our Editorial Policies and the Editorial Policy Checklist.

Statistics

For all statistical analyses, confirm that the following items are present in the figure legend, table legend, main text, or Methods section.

n/a	Confirmed	
☐	☒	The exact sample size (*n*) for each experimental group/condition, given as a discrete number and unit of measurement
☐	☒	A statement on whether measurements were taken from distinct samples or whether the same sample was measured repeatedly
☐	☒	The statistical test(s) used AND whether they are one- or two-sided<br>*Only common tests should be described solely by name; describe more complex techniques in the Methods section.*
☐	☒	A description of all covariates tested
☐	☒	A description of any assumptions or corrections, such as tests of normality and adjustment for multiple comparisons
☐	☒	A full description of the statistical parameters including central tendency (e.g. means) or other basic estimates (e.g. regression coefficient) AND variation (e.g. standard deviation) or associated estimates of uncertainty (e.g. confidence intervals)
☐	☒	For null hypothesis testing, the test statistic (e.g. *F*, *t*, *r*) with confidence intervals, effect sizes, degrees of freedom and *P* value noted<br>*Give P values as exact values whenever suitable.*
☒	☐	For Bayesian analysis, information on the choice of priors and Markov chain Monte Carlo settings
☒	☐	For hierarchical and complex designs, identification of the appropriate level for tests and full reporting of outcomes
☒	☐	Estimates of effect sizes (e.g. Cohen's *d*, Pearson's *r*), indicating how they were calculated

Our web collection on statistics for biologists contains articles on many of the points above.

Software and code

Policy information about availability of computer code

Data collection	REDCap was used for data capturing during the main trial.
Data analysis	We used NanoCLUST (version 1.0dev) [https://github.com/Clinical-Infection-Research-UoSheffield/nf-core-nanopath] to analyze the raw sequence data. We ran NanoCLUST using Nextflow (version 21.10.6). We used R (version 4.4.0) and RStudio (version 2024.04.1+748) for data cleaning, visualization and statistical analyses. The following packages were used in R: Tidyverse (version 2.0.0), phyloseq (version 1.48.0), decontam (version 1.20.0), vegan (version 2.6.4), MaAsLin2 (version 1.18.0). We conducted unsupervised clustering in mothur (version 1.44.0) using Dirichlet's Multinomial Mixtures model. The codes used for microbial community analysis in R are available on Github [https://github.com/Baksso/Azimic/blob/main/MIC2_gut_mycobiota_analysis.Rmd].

For manuscripts utilizing custom algorithms or software that are central to the research but not yet described in published literature, software must be made available to editors and reviewers. We strongly encourage code deposition in a community repository (e.g. GitHub). See the Nature Portfolio guidelines for submitting code & software for further information.

Data

Policy information about availability of data

All manuscripts must include a data availability statement. This statement should provide the following information, where applicable:

- Accession codes, unique identifiers, or web links for publicly available datasets
- A description of any restrictions on data availability
- For clinical datasets or third party data, please ensure that the statement adheres to our policy

The sequence data generated in this study has been deposited in the SRA under the bioproject accession PRJNA1129542 [https://www.ncbi.nlm.nih.gov/bioproject/PRJNA1129542]. The data on bacterial community types of the samples used in this analysis is available on figshare at this link https://doi.org/10.6084/m9.figshare.29357054. Additional demographic data on the study participants is available upon request. The data in this study has been collected following provision of informed consent under the prerequisite of strict participant confidentiality. Access can be requested through the Gambia Government/MRC Joint Ethics Committee. The review process and release of data will be facilitated by MRC Unit The Gambia (http://www.mrc.gm/) through the Head of Governance Mr Dembo Kanteh (Dembo.Kanteh@lshtm.ac.uk) and the corresponding author Prof Anna Roca (aroca@mrc.gm). The scientific merit of the request will be evaluated by the Scientific Coordinating Committee at MRCG at LSHTM. Response would be given within 8 weeks. All other data are available within the Article and Supplementary files.

Research involving human participants, their data, or biological material

Policy information about studies with human participants or human data. See also policy information about sex, gender (identity/presentation), and sexual orientation and race, ethnicity and racism.

Reporting on sex and gender	Sex of the children were determined at birth. We assessed sex representation between trial arms at baseline to ensure similar representation. This information is provided in table 1. In addition, we also included sex as a biological variable in our analysis and reported the results in the manuscript. We also discussed the relevance of our findings with regards to sex within the context of intrapartum azithromycin intervention.
Reporting on race, ethnicity, or other socially relevant groupings	This manuscript contains data on maternal age and ethnicity. Age was provided by participants through their official identity cards and ethnicity by self-reporting. Information on participants ethnicity and age are summarized in table 1 in the manuscript.
Population characteristics	The children included in this analysis were selected from the Gambian cohort of the PregnAnZI-2 trial (ClinicalTrials.org NCT03199547). PregnAnZI-2 was a phase-III, double-blind, randomised, placebo-controlled trial in which 11,983 women from The Gambia and Burkina Faso were randomized to receive a single dose of 2g of oral azithromycin or placebo (ratio 1:1) during labour. Details of inclusion and exclusion criteria are available in the study protocol provided with the manuscript and also cited in the main manuscript text in the methods. The primary objective of the trial was to evaluate the impact of the intervention on neonatal sepsis and death as well as maternal infections. The trial showed no effect on neonatal sepsis or death though other infections, including skin infections, were reduced in the azithromycin arm. Also, the intervention reduced maternal infections including mastitis. The children selected for this analysis were healthy term-born babies who were followed from birth to the age of three years. The mothers of the children were women between the ages of 16 and 42 years, who lived in peri urban settlements in the coastal region of the Gambia.
Recruitment	Women who participated in the PregnAnZI-2 trial were consented and recruited during normal antenatal visits. Participation was entirely voluntary. In the Gambia, recruitment took place at two major health centers within the greater Banjul area which represents a typical peri urban setting in sub Saharan Africa. The women were screened by clinicians for any health conditions that may bias the results of the study. Also, women who were on antibiotics in the preceding week before administration of the intervention were excluded. The study was double-blinded and the intervention randomized and placebo-controlled making a robust design to minimize different biases. There may be some risk of selection bias in the sense that women who attended more antenatal clinics and delivered in the health centers are more likely to be consented and recruited. However, we do not anticipate much effect from this as 90% of pregnant women in The Gambia attend antenatal clinics at least once and 70% at least four times. Also the study catchment area is a peri-urban settlement in the coastal region, which may have some differences with remote settlements far inland. Therefore, our results may not be entirely generalizable to all populations, especially, typical rural populations.
Ethics oversight	This study was approved by the Gambia Government-MRC Gambia joint ethics committee, and the London School of Hygiene and Tropical Medicine Ethics Committee.

Note that full information on the approval of the study protocol must also be provided in the manuscript.

Field-specific reporting

Please select the one below that is the best fit for your research. If you are not sure, read the appropriate sections before making your selection.

- [x] Life sciences
- [ ] Behavioural & social sciences
- [ ] Ecological, evolutionary & environmental sciences

For a reference copy of the document with all sections, see nature.com/documents/nr-reporting-summary-flat.pdf

Life sciences study design

All studies must disclose on these points even when the disclosure is negative.

Sample size	The sample size was originally calculated for gut microbiota analysis. We chose to work with the same set of samples because we intended to link our findings with the results we obtained from the gut microbiota analysis. The sample size calculation was based on power to detect at least 10% difference in the top 10 operational taxonomic units (OTUs) and 20% difference in the next 10 OTUs in the gut microbiota using a sample size and power calculation tool for case-control microbiome study design developed by Mattiello et al. We used the gut microbiome dataset from the human microbiome study embedded in the tool. With a sample size range of 30 -70 per group, we estimated power by Monte Carlo simulations with 100 replications using the top 50 OTUs from the dataset. A sample size of 45 per group at each time-point had over 90% power to detect these differences. Though we expect higher interpersonal variations with the gut mycobiota than the gut microbiota, our analysis factored this by exclusion of outliers in comparisons of community composition and differential taxon abundance. The remaining samples used in each of these analyses are shown in the respective results.
Data exclusions	In our analysis of community composition and differential taxon abundance, samples with only one or two taxa were excluded as this would skew the data and thus affect the results. Such samples where regarded as outliers.
Replication	We have provided a detailed explanation of the methods applied in the study and provided the raw sequences and analysis codes used. We have also provided other data that were generated from our previous study which was used in the current analysis. We did repeat the analysis multiple times and were able to reproduced the same results.
Randomization	The main trial included individual randomization to treatment or placebo arms (ratio 1:1).
Blinding	The trial was double-blinded with both participants and the investigators blinded to the randomization allocation. This posthoc analysis was conducted after opening the trial code. However, the investigators were blinded during sample selection and laboratory analysis but not at the final analysis stage as information on trial arm allocations was needed for the final analysis.

Reporting for specific materials, systems and methods

We require information from authors about some types of materials, experimental systems and methods used in many studies. Here, indicate whether each material, system or method listed is relevant to your study. If you are not sure if a list item applies to your research, read the appropriate section before selecting a response.

Materials & experimental systems

n/a	Involved in the study
☒	☐ Antibodies
☒	☐ Eukaryotic cell lines
☒	☐ Palaeontology and archaeology
☒	☐ Animals and other organisms
☒	☐ Clinical data
☒	☐ Dual use research of concern
☒	☐ Plants

Methods

n/a	Involved in the study
☒	☐ ChIP-seq
☒	☐ Flow cytometry
☒	☐ MRI-based neuroimaging

Plants

Seed stocks	*Report on the source of all seed stocks or other plant material used. If applicable, state the seed stock centre and catalogue number. If plant specimens were collected from the field, describe the collection location, date and sampling procedures.*
Novel plant genotypes	*Describe the methods by which all novel plant genotypes were produced. This includes those generated by transgenic approaches, gene editing, chemical/radiation-based mutagenesis and hybridization. For transgenic lines, describe the transformation method, the number of independent lines analyzed and the generation upon which experiments were performed. For gene-edited lines, describe the editor used, the endogenous sequence targeted for editing, the targeting guide RNA sequence (if applicable) and how the editor was applied.*
Authentication	*Describe any authentication procedures for each seed stock used or novel genotype generated. Describe any experiments used to assess the effect of a mutation and, where applicable, how potential secondary effects (e.g. second site T-DNA insertions, mosiacism, off-target gene editing) were examined.*
